# Evaluating Unimodal and Multimodal Tracking Strategies for the Reconstruction of Language-related White Matter Tracts

**DOI:** 10.1007/s00062-025-01601-9

**Published:** 2025-12-10

**Authors:** Lydia Marleen Schilling, Anna-Lena Ciesla, Julia My Van Kube, Peter Dechent, Christian Heiner Riedel, Nicole E. Neef

**Affiliations:** 1 Department of Diagnostic and Interventional Neuroradiology, Universitätsmedizin Göttingen, Göttingen, Germany; 2 Heart & Brain Center, Department of Cognitive Neurology, MR Research in Neurosciences, Universitätsmedizin Göttingen, Göttingen, Germany

**Keywords:** Diffusion MRI, Functional MRI, White matter tracts, Language, Preoperative mapping

## Abstract

**Purpose:**

Accurate reconstruction of language-related white matter pathways is essential for preoperative planning in brain surgery. While functional (f) MRI activation is often used to guide diffusion (d)MRI tractography, advanced automated protocols rely instead on subcortical anatomical priors. In this study, we evaluate the robustness of anatomically informed protocols without fMRI and compare them with fMRI-guided approaches.

**Methods:**

Twenty healthy adults (aged 18–32) underwent fMRI during a language task and dMRI on a 3T MRI scanner. Six language-associated fiber bundles were reconstructed with XTRACT using unimodal and multimodal protocols. Reconstruction similarity was assessed using cross-correlations within-subjects, within-cohort, and across-cohorts. Normalized streamline counts served as a proxy for connectivity, and *t*-tests were used to quantify differences between tracking protocols.

**Results:**

Protocols using anatomically informed subcortical seed and target masks, as well as their combination with functional masks, yielded higher agreement and greater normalized streamline counts than the fMRI-only protocol. The combined approach showed an additional advantage for reconstructing parieto-temporal white matter tracts.

**Conclusions:**

These comparisons underscore how protocol choice shapes the reconstruction of language pathways and highlight the need to evaluate these tractography strategies in clinical cohorts.

**Supplementary Information:**

The online version of this article (10.1007/s00062-025-01601-9) contains supplementary material, which is available to authorized users.

## Introduction

Diffusion (d)MRI tractography enables non-invasive, in vivo mapping of white matter tracts and aids preoperative diagnostics for various brain pathologies, including tumors, epilepsy, and deep brain stimulation planning. In language-sensitive regions, functional (f)MRI often guides tractography to refine functional anatomy [1–4]. However, language fMRI is time-consuming, task-dependent, and requires patient compliance [5–7]. Minimizing its use could make preoperative neuroradiologic assessment more efficient and consistent. This study tests whether a previously published automated tractography protocol without fMRI-guided seed and target masks [8] performs comparably to, or better than, conventional fMRI-informed approaches for the purpose of language mapping.

From a clinical perspective, maximizing tumor resection is associated with improved outcomes in both low- [9] and high-grade gliomas [10, 11], though it must be carefully balanced against the risk of prospective cognitive deficits [12, 13]. Tractography has become a cornerstone of this onco-functional balance [14], enabling patient-specific mapping of white matter tracts [15] and overcoming the anatomical constraints of traditional cadaveric dissection [16–18]. It informs surgical planning by identifying critical white matter pathways involved in motor, sensory, cognitive, speech and language functions [19, 20]. Tractography helps in delineating safe surgical corridors, defining resection boundaries, and guiding surgical trajectories [21, 22], while also supporting intraoperative functional mapping through direct electrical stimulation or neurophysiological monitoring [23–25]. Beyond tumor surgery, tractography also contributes to other neurosurgical applications. For example, it helps characterize connectivity patterns associated with clinically effective electrode placements in patients with Parkinson’s disease undergoing subthalamic nucleus deep brain stimulation [26], and assists in optimizing the placement of stereo-EEG electrodes for epilepsy diagnostics using intracranial EEG [27]. White matter tracts are now increasingly recognized as essential not only for functional preservation but also for facilitating post-surgical neuroplasticity, particularly in multifunctional brain regions [28–30]. dMRI tractography plays a central role in guiding clinical decision-making across various neurological domains.

fMRI is commonly used to localize eloquent cortex for surgical planning, with recent guidelines outlining best clinical practices [7, 31]. The arcuate fasciculus, a key language pathway linking frontal, parietal, and temporal regions, including Broca’s and Wernicke’s areas, is often reconstructed using dMRI tractography seeded from fMRI-identified language areas [1, 32–34]. However, both fMRI language mapping and dMRI tractography are resource-intensive. Language fMRI depends on patient compliance and is time-consuming, while tractography requires specialized expertise and is highly sensitive to user-defined parameters [35]. Additionally, commercial software varies in quality and often lacks robust algorithms to account for pathological tissue, limiting reliability in clinical use [36]. These limitations highlight the need for standardized, robust, and accessible tractography methods, whether guided by fMRI or not, to support broader clinical integration.

To overcome the limitations of conventional tractography workflows, recent efforts have focused on automated and standardized approaches. One automated pipeline for reconstructing language-related tracts, based on atlas-derived anatomical criteria and clinical diffusion protocols, has shown strong agreement with expert segmentations and alignment with task-based fMRI [34]. Similarly, XTRACT, a publicly available tool integrated into FSL (https://fsl.fmrib.ox.ac.uk/fsl/docs/#/), provides robust and reproducible tractography protocols for the adult human brain [8]. The tool offers robust extraction of white matter tracts using data from large-scale datasets such as the Human Connectome Project and UK Biobank. These developments mark important progress toward reliable and clinically scalable tractography.

The aim of this study was to compare two standardized tractography protocols for reconstructing language-related white matter tracts: a unimodal approach using only diffusion MRI, and a multimodal approach guided by functional MRI. For the unimodal method, we applied the standard XTRACT protocol, which places seed and target masks within white matter structures [8]. For the multimodal method, we used anatomically constrained, fMRI-derived seed masks placed in cortical regions. We also evaluated a combined approach that integrates XTRACT-defined subcortical masks with fMRI-guided cortical seed masks. We focused on six major language structures: the frontal aslant tract (FAT), arcuate fasciculus (AF), superior longitudinal fascicules III (SLF3), middle longitudinal fasciculus (MdLF), inferior longitudinal fasciculus (ILF), and uncinate fasciculus (UF) [8, 37]. The FAT connects the inferior frontal gyrus (IFG) with medial frontal regions and is primarily implicated in speech initiation and verbal fluency [38, 39]. The AF and SLF3 form the dorsal language pathway [40–42], linking frontal parietal and temporal regions to support sound-to-articulation mapping [43], complex syntax processing [44], and semantic [45]. The MdLF, ILF, and UF form the ventral pathway [40, 41], which has been associated in particular with semantic processing and language comprehension [43, 46], though it supports other functions such as episodic memory, associative learning, and social and emotional functioning [47, 48] as well.

Language fMRI activation maps were obtained using a word decision task that contrasted the processing of compound words with the processing of non-lexical, rhyming pseudowords. The compound > rhyme contrast highlights regions involved in semantic processing by comparing meaningful stimuli (compound words) with phonologically similar but meaningless ones. Conversely, the rhyme > compound contrast identifies regions involved in phonological processing and articulatory mapping, as it emphasizes sound-based decision-making in the absence of semantic content. This task design also controls for domain-general cognitive demands [49] such as working memory, attention, and response inhibition, as well as for sensory and motor aspects of task performance [31]. Prior studies have shown that lexical versus sublexical contrasts reliably engage language-selective cortical regions involved in higher-order language processing [50–52].

By comparing tractography outputs across unimodal and multimodal conditions, we aimed to assess how functional seeding influences the delineation of these distinct language-related fiber bundles. Our goal was to inform more efficient and differentiated approaches for preoperative planning. If validated, our findings could help optimize preoperative imaging workflows and make structural language mapping more accessible in clinical practice.

## Methods

### Participants

Twenty healthy volunteers (10 females; age 18–32 years, mean 24 years) participated. Exclusion criteria included MRI contraindications, neurological or psychiatric disorders, chronic diseases requiring medication, drug abuse, and current pregnancy or breastfeeding. All were native German speakers with normal or corrected-to-normal vision. All were right-handed (Edinburgh Inventory [53]; laterality quotient 70–100). The study was approved by the Ethics committee of the University Medical Center Göttingen and was in accordance with the guidelines of the Declaration of Helsinki. All participants gave written informed consent and obtained an expense allowance for taking part in this study.

## Language Mapping Paradigm

**Fig. 1:**
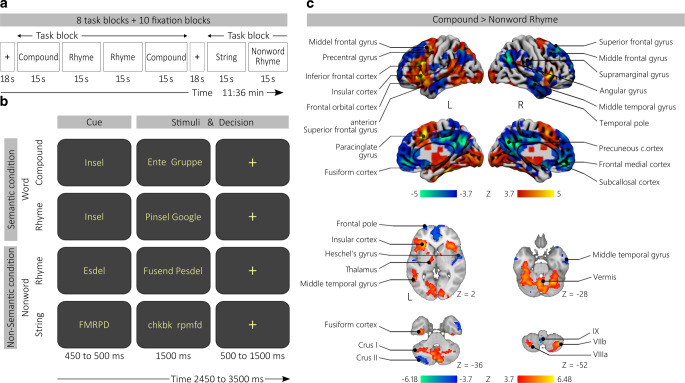
Illustration of the functional MRI. **a** paradigm, **b** example trials, **c** group average functional map

Language maps were derived using a mixed event-related paradigm (Fig. 1a) targeting two semantic (compound, rhyme) and two non-semantic (pseudoword rhyme, consonant string) language conditions. Each trial consisted of a cue, a stimulus, and a decision phase during which participants responded with their left or right thumb on a button box. Correct response positions (left/right) were pseudorandomized across trials. Participants first saw a word or nonword cue, followed by two response options, one target and one distractor. Figure 1b shows a sample instance from each condition. In the compound condition, participants selected the word that formed a valid compound with the cue, e.g., *Insel (island)* → *Ente (duck)* instead of *Gruppe (group)*. In the rhyme condition, they chose the word that rhymed with the cue, e.g., *Insel* → *Pinsel* (*brush*) instead of *Google (Google)*. For pseudoword rhyming, nonword cues, e.g., *Esdel* [′ɛz.dəl] required selecting a rhyming nonword, e.g., *Pesdel* [′pɛz.dəl] instead of *Fusend *[′fu:.zənd]. In the consonant string condition, cues were vowel-free letter strings, e.g., *FMRPD*, and participants identified the string with the same consonants in a different order, e.g., *rpmfd* instead of *chkbk*. Nonwords were matched to word stimuli on syllable count, phonetic complexity, and phonotactics, generated using Wuggy [54]. Consonant strings were created manually or with ChatGPT (GPT‑3.5, https://chat.openai.com). The paradigm comprised 640 trials in total (160 per condition). Stimuli were presented in trains of five trials from a single condition, with two trains per condition and two conditions randomized within each block (Fig. 1a). Trial onset intervals were jittered: 2450–3450 ms (in 250 ms steps) for the compound, rhyme, and pseudoword rhyme conditions; 2500–3500 ms for the consonant string condition. Each block lasted approximately one minute, followed by an 18-second pause. Each run included 8 task blocks and 10 fixation blocks, lasting 11 min and 36 s in total. Each participant completed four runs.

## Data Acquisition

MRI imaging was conducted in a Siemens 3 T Magnetom Prisma Fit using a 64-channel head coil for signal reception. Initially, structural whole-brain T1WI MRI were recorded using a non-selective inversion-recovery 3D turbo fast low-angle shot sequence (TR = 1900 ms, TE = 2.26 ms, TI = 900 ms, flip angle = 9°, FoV = 256 mm, 7/8 Fourier phase encoding) at 1 mm^3^ isotropic spatial resolution. Functional data was acquired with a gradient-echo echo planar imaging (EPI) sequence (TR = 1500 ms, TE = 30 ms, flip angle = 72°, parallel acquisition factor = 2, simultaneous multi-slice factor = 3, FoV = 208 mm, 63 axial slices) with an in-plane resolution of 2 × 2 mm^2^, a distance factor of 10% and a 104 × 104 acquisition matrix. We acquired 902 volumes per run. Diffusion-weighted MRI was performed using a spin-echo EPI sequence with the following imaging parameters: voxel size 2 × 2 × 2 mm^3^, TE = 82 ms, TR = 7.5 s, FoV = 104 × 104, 64 axial slices covering the whole brain, no inter-slice gap. Diffusion-weighted MRI data were acquired for 64 diffusion-encoding gradient directions with a b-value of 1000 s/mm^2^. In addition, one anatomical reference image without diffusion-weighting (b-value = 0 s/mm^2^) was obtained for offline motion correction. The dMRI acquisition was accelerated using partial Fourier imaging (factor 6/8) and parallel imaging (GRAPPA) with an acceleration factor of 2. Fat saturation was applied using a spectral saturation pulse. The dMRI sequence took 8 min 32 s. To correct for susceptibility-induced distortions, an additional spin-echo EPI image without diffusion-weighting and with reversed phase-encoding direction was acquired for field unwarping using the topup method [55].

## Preprocessing

T1WI were segmented using FastSurfer [56]. Functional data were analyzed with FSL v6.0 using the FEAT pipeline [57, 58]. Preprocessing included motion correction, 5 mm FWHM spatial smoothing, high-pass filtering (cutoff = 90 s), co-registration to the anatomical image, and normalization to the MNI152 T1 template. First-level models included the four conditions convolved with the hemodynamic response function, with temporal derivatives as nuisance regressors. Two contrasts were computed: compound > nonword rhyme and nonword rhyme > compound. Second-level analysis averaged these contrasts across the four runs. The resulting maps were used to examine cortical correlates of language processing (Fig. 1c); other conditions pertain to a separate study. Statistical inference used a cluster-forming threshold of Z > 3.1 and a family-wise error-corrected extent threshold of *p* < 0.05 [59].

Diffusion data were first denoised and corrected for Gibbs ringing artifacts using the *dwidenoise* and *mrdegibbs* commands in the MRtrix package (https://github.com/MRtrix3/mrtrix3, [60]). Further preprocessing was carried out with the FMRIB Software Library (FSL, http://www.fmrib.ox.ac.uk/fsl), including correction for susceptibility-induced distortions, eddy currents, and head motion using *topup* and *eddy*. Brain extraction was then performed, and standard DTI contrast maps were computed. All processed datasets were visually inspected to ensure the absence of artifacts from acquisition or preprocessing.

## Fiber Reconstruction and Quantification

**Fig. 2:**
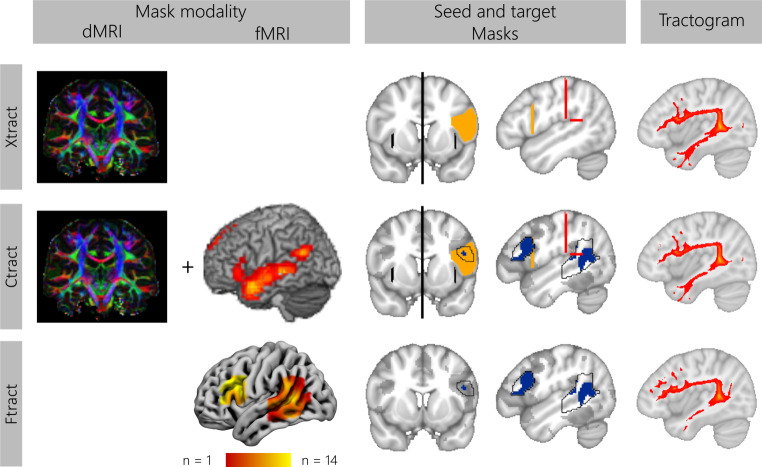
Illustration of the three tracking protocols for the left arcuate fasciculus. Xtract is an automated unimodal protocol using a seed mask in the white matter beneath the inferior frontal gyrus (orange), and two target masks (red), one beneath the supramarginal gyrus, and another beneath the superior temporal and middle temporal gyrus. Ctract is a combined multimodal protocol that additionally includes fMRI-guided cortical seed and target masks (dark blue). The second row, column two shows a subject-level activation map (compound > nonword rhyme), on the cortical surface. The coronal and sagittal brain slices next to it display the seed and target voxels (dark blue) respectively. The bottom row displays a 3d brain rendering with the voxel-wise percentage overlap maps of the functional seed and target masks. Ftract uses only fMRI-guided masks. Dark lines in the brain slices indicate the functional partitions [51, 52] used to anatomically constrain the functional-guided masks. Black represents exclusion masks. The right column illustrates the resulting fiber tract maps for each approach respectively

Three tractography approaches were evaluated in this study: (a) Xtract, an automated unimodal method using diffusion MRI alone, as implemented in the XTRACT toolbox [8]; (b) Ftract, a multimodal method guided by fMRI-derived seed and target masks; and (c) Ctract, a combined approach that integrates seed and target masks from both Xtract and Ftract to enhance anatomical and functional coverage. Figure 2 illustrates these tracking protocols using the AF as an example.

*Unimodal automated fiber quantification (Xtract): *Standardized automated fiber quantification was performed as previously described [8], using the XTRACT toolbox and probabilistic tractography was conducted based on predefined standard-space protocols [61]. Each white matter bundle was reconstructed using a specific combination of seed, target, and stop masks [8]. These masks were previously developed based on the MNI152 standard space anatomical atlases and refined through expert consensus by five specialists involved in creating the XTRACT protocol [8]. All protocols include the midline sagittal plane as an exclusion mask to restrict tracking to the ipsilateral hemisphere. As implemented in XTRACT (FSL version 6.0.7.16), two seeding strategies were applied: (a) standard single-region of interest (ROI) seeding for FAT, SLF3, AF, and UF; and (b) a reverse-seeding approach, where seed and target masks were swapped and resulting path distributions combined for MdLF and ILF (https://git.fmrib.ox.ac.uk/fsl/xtract). All masks were warped to each subject’s native space, and the resulting tractography outputs were resampled back into standard space. All protocols used default *probtrackx2* parameters: a curvature threshold of ± 80°, maximum 2000 streamline steps, 1% subsidiary fiber volume threshold, randomized seeding in regions of crossing fibers, loop-checking, and no minimum length constraint. Step sizes of 0.5 mm and 0.2 mm were used. The tract distributions were then normalized based on the total number of valid streamlines, i.e., those not rejected by the inclusion or exclusion masks.

*Multimodal fMRI-based fiber quantification (Ftract): *fMRI-guided fiber tracking was also conducted using the *xtract* function implemented in FSL. To integrate functional information into the tractography protocols, we used individual FEAT outputs from the language task contrasts (compound > nonword rhyme or nonword rhyme > compound). The resulting thresholded and binarized activation maps were multiplied with anatomically plausible cortical seed and target masks, ensuring that only voxels that were both functionally active and located within relevant cortical regions were included in the Ftract protocol. For example, in the reconstruction of the AF, only activated voxels within masks of the inferior frontal gyrus and the posterior temporal lobe were retained as cortical seeds and targets, consistent with the tract’s known terminations [62]. This procedure was applied individually for each participant, allowing functional localization to refine tractography seeding while maintaining anatomical specificity. Anatomical masks were derived from two sources. In the following, full labels and their corresponding abbreviations are provided here for reference. First, from an fMRI-guided partitioning of the cortex [51, 52], we included the inferior frontal gyrus encompassing pars opercularis and pars triangularis (IFG), the inferior frontal gyrus pars orbitalis (IFGorb), the anterior temporal region (AntTemp), and the posterior temporal region (PostTemp). Second, we included additional regions based on morphometric partitions from the Harvard-Oxford Cortical Structural Atlas as implemented in FSL, including the inferior frontal gyrus pars opercularis (IFGop), superior frontal gyrus (SFG), the precentral gyrus (pCG), supplementary motor area (SMA), the anterior and posterior divisions of the supramarginal gyrus (aSMG, pSMG), angular gyrus (AG), superior temporal gyrus (STG), and the temporal pole (TP). For each tract, these masks were used to constrain seed and target regions as follows. The seed region for the FAT was constrained by the SFG and SMA, and the target region by the IFG. For the AF, the seed region was constrained by the IFG and the target by PostTemp. The seed region for the SLF3 was constrained by the pCG and IFGop, with targets in the aSMG and pSMG. For the MdLF, the seed mask included STG and AG, while the target was constrained to the TP. The ILF was tracked using a PostTemp seed and AntTemp target. Lastly, the UF was seeded in the AntTemp and targeted to the IFG and IFGorb. Tract-specific individual cortical masks were stored within a dedicated protocol folder, and specified via the −*p* option of the *xtract* function. The analyses were performed using the default parameters, as outlined in the preceding description of the Xtract protocol. Supplementary Table 7 provides an overview of the anatomically-constraining masks and the associated functional overlap maps, illustrating the proportions of voxels contributing to each tract in the Ftract protocol across participants.

*Multimodal combined fiber quantification (Ctract)*: For the combined protocol, the anatomically defined subcortical seed and target masks from Xtract were migrated with the functionally informed cortical masks from Ftract into a dedicated protocol folder. Therefore, the respective Xtract and Ftract seed masks were subtracted and binarized with *fslmaths*. For the standard single-ROI seeding protocols the target mask was simply added as an additional target mask. For the reverse-seeding approach the Xtract and Ftract target masks were subtracted and binarized with *fslmaths *to generate a single target mask. This ensured that both anatomical and functional information contributed to defining the tract-specific regions of interest. Fiber tracking was then again performed with the *xtract* function in FSL, allowing anatomical priors and individual functional activation to jointly constrain the reconstruction.

To delineate tracts, we applied a 0.5%-of-maximum threshold to the normalized path distribution of each individual tract. The resulting maps were then binarized. To visualize the anatomical location and consistency of the reconstructed tracts in the current sample, the binarized masks were averaged across participants to generate voxel-wise percentage overlap maps indicating the proportion of subjects in whom each tract was present.

## Statistical Analyses

Our aim was to determine whether Xtract, Ftract, and Ctract yield similar or different tract reconstructions. To this end, we calculated cross-correlations on the thresholded binarized tract maps to assess the spatial overlap between reconstructions across protocols and subjects. Specifically, we calculated (1) *within-subject* similarity, (2) *within-cohort* (between-subjects) similarity, and (3) *across-cohort* similarity. All correlation analyses were conducted using the FSL function *fslcc*, applied to normalized tract-density maps thresholded at 0.5% of the maximum and binarized [8]. Because correlation coefficients are not normally distributed and are not measured on an interval scale, we applied a Fisher *Z*-transformation to all correlation values prior to group-level statistical analyses. This transformation stabilizes variance and renders the values more suitable for parametric testing [63].

1. We quantified *within-subject* similarity by correlating the thresholded, normalized path distributions in MNI space for Xtract-Ctract, Xtract-Ftract, and Ctract-Ftract, separately for each tract and subject. To facilitate interpretation of correlation strength, we followed the benchmark established by Warrington et al. [8], who compared each tract between the Human Connectome Project (HCP) cohort [64] and more typical data from the UK Biobank [65]. Consistent with their findings, *within-subject* cross-correlations of *Z* ≥ 1.1 (*r* ≥ 0.80) [8] were taken to indicate very good to strong agreement.
2. We evaluated *intra-cohort* (between-subject) similarity by computing pairwise correlations across all subject pairs for each tract. The benchmark reference value for *intra-cohort* similarity was *Z* ≥ 0.56 (*r* ≥ 0.51) [8].
3. *Across-cohort* similarity was assessed by correlating each individual’s tract with the corresponding HCP tract from the Xtract toolbox [8]. The benchmark reference value for a*cross-cohort* similarity was *Z* ≥ 0.44 (*r* ≥ 0.41) [8].

For comparison with a large normative dataset, similarity values were referenced to those reported by Warrington and colleagues [8] based on the HCP sample. Although the raw HCP diffusion data are publicly available, tract-specific benchmark values are not. As a result, we used the published summary statistics for the full set of 46 Xtract tracts as reference values, rather than tract-specific similarity metrics for the 12 language-related tracts analyzed in the present study.

Fisher Z‑transformed correlation values for each tract were then entered into one-sample *t*-tests to assess the stability of tract reconstruction relative to its benchmark value. In addition, paired *t*-tests were performed for each tract-protocol pair to evaluate differences between tracking protocols. All *p*-values were adjusted for multiple comparisons using the Holm-Bonferroni correction.

Finally, we note that high similarity between tract maps does not, by itself, guarantee accurate reconstruction of the underlying fiber bundle. As a complementary indicator of reconstruction quality, we use the normalized streamline counts, with higher values indicating stronger bundles. For each participant and each seed-target pair, we computed a normalized streamline index (NSI) from the number of sample streamlines leaving the seed that reached the target (waytotal). Because this raw count scales with seed size, we normalized by the theoretical maximum number of samples (*N*_*samples*_ × *V*_*seed*_) and defined

$$NSI=\frac{\log \left(\textit{waytotal}\right)}{\log \left(N_{\textit{samples}}\times V_{seed}\right)}$$

The logarithmic scaling renders the index approximately normal across participants and constrains it to [0.1], with larger values indicating stronger seed-target connections [66, 67]. For statistical comparison, NSI values were analyzed with paired *t*-tests to assess differences between Xtract and Ctract, Xtract and Ftract, and Ctract and Ftract. All *p*-values were adjusted for multiple comparisons using the Holm-Bonferroni correction.

## Results

### Within-subject Similarity

**Fig. 3:**
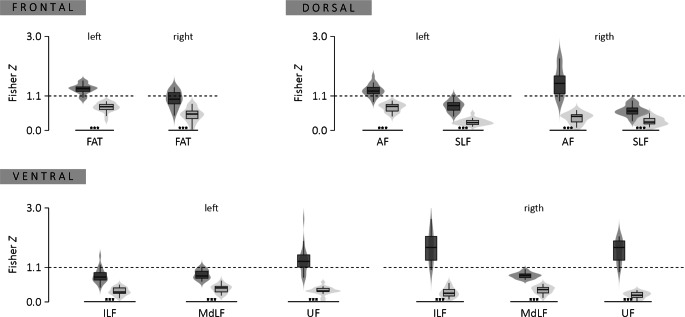
*Within-subject* similarity in cross-correlations between tracking protocols. Agreement between Xtract and Ctract (dark gray) was consistently higher than between Xtract and Ftract (light gray). The dotted line marks the benchmark threshold *Z* = 1.1 [8]. Statistical significance was assessed using paired *t*-tests with Holm-Bonferroni correction (***p < 0.001)

We assessed similarity among tract reconstructions from three tracking protocols. For each participant and tract, we cross-correlated the Xtract reconstruction with its Ctract and Ftract counterparts (Fig. 3). Across all tracts, average Fisher *Z*-transformed correlation coefficients were higher for Xtract-Ctract (*M* = 1.14, *SD* = 0.10) than for Xtract-Ftract (*M* = 0.41, *SD* = 0.06; paired *t*-test: *t*(19) = 25.80, *p* < 0.001), a pattern consistently observed for each tract (Fig. 3, Supplementary Table 3). Compared with the benchmark for *within-subject* similarity, the Xtract-Ftract comparison yielded consistently lower agreement. In contrast, Xtract-Ctract showed a mixed pattern: high agreement for the left and right AF and UF, left FAT, and right ILF; comparable agreement for the right FAT; and lower agreement for bilateral MdLF, SLF3 and left ILF (Fig. 3, Supplementary Table 3).

## Intra-cohort Similarity

**Fig. 4:**
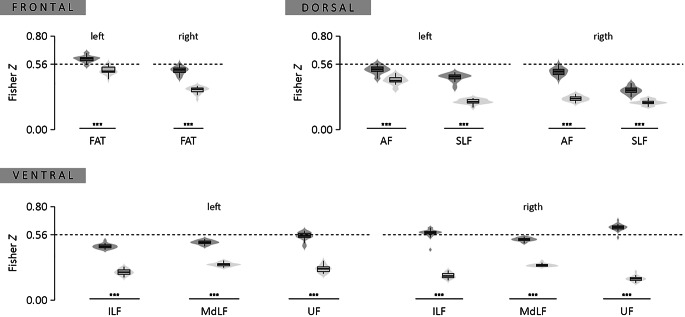
*Between-subjects* similarity for the cross-correlation between protocols. Agreement between Xtract and Ctract (dark gray) was consistently higher than between Xtract and Ftract (light gray). The dotted line marks the benchmark threshold *Z* = 0.56 [8].***Paired *t*-tests, *p* < 0.001, Holm-Bonferroni correction

We also assessed *intra-cohort* similarity by examining pairwise correlations of tract reconstructions across participants for each protocol. *Intra-cohort *comparisons were higher for Xtract-Ctract with Fischer *Z* = 0.51 (*SD* = 0.01) than for Xtract-Ftract with Fisher *Z* = 0.29 (*SD* = 0.01; paired *t*-test: *t*(19) = 82.18, *p* < 0.001), a pattern consistently observed for all tracts (Fig. 4, Supplementary Table 4). Compared with the benchmark value, Xtract-Ctract *intra-cohort* correlations were lower overall, *t*(19) = −18.56, *p* < 0.001. Specifically, reduced values were found for the left and right AF, SLF and MdLF, left ILF and UF and the right FAT, whereas the left FAT and right ILF and UF showed higher correlations (Fig. 4, Supplementary Table 4). By contrast, Xtract-Ftract *intra-cohort* correlations were markedly lower than those of the benchmark value (*t*(19) = −197.79, *p* < 0.001), a pattern evident across all tracts (Fig. 4, Supplementary Table 4).

## Across-cohort Similarity

**Fig. 5:**
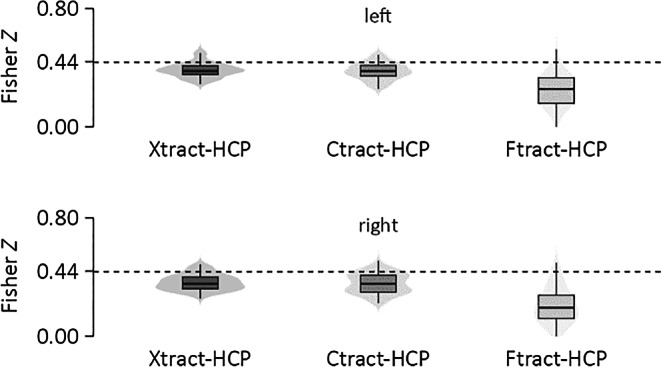
*Across-cohort *similarity for the cross-correlation between each protocol and the HCP template tract. Agreement for Xtract (*dark gray*) and Ctract (*medium gray*) was closer to the HCP template than for Ftract (*light gray*). The dotted line indicates the average *across-cohort* robustness between HCP and UK Biobank. ***Paired *t*-tests, *p* < 0.001, Holm-Bonferroni corrected

Finally, to assess *across-cohort* robustness, we compared HCP tracts with reconstructions from our three protocols. For each tract and participant, the HCP reconstruction was cross-correlated with its Xtract, Ctract, and Ftract counterparts. Mean correlations were slightly lower for Xtract-HCP (Fischer *Z* = 0.37, *SD* = 0.02) and Ctract-HCP (Fischer *Z* = 0.37, *SD* = 0.02), and lowest for Ftract-HCP (Fisher *Z* = 0.23, *SD* = 0.04). Figure 5 and Supplementary Table 5 show the average *across-cohort* correlations by protocol and hemisphere. All *t*-tests indicate significant lower correlations. When considering Xtract alone, *across-cohort* correlations were lower in this study, which focused exclusively on language-related tracts, compared with the benchmark value derived from all 46 Xtract tracts (*t*(19) = −12.83, *p* < 0.001).

## Normalized Streamline Counts

**Fig. 6:**
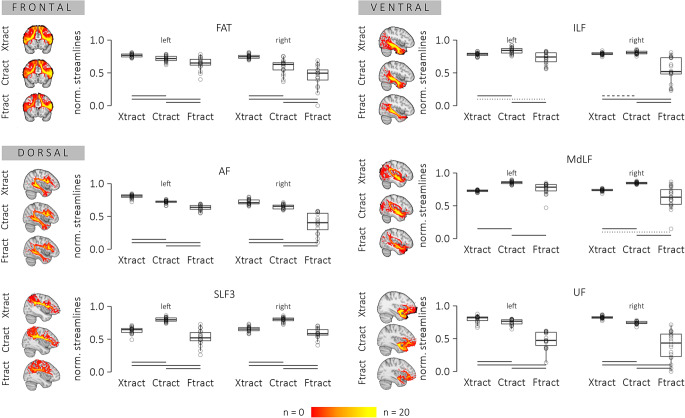
Overlap maps and comparison of normalized streamlines as a measure of connection strength. The left column in each panel displays tract overlap maps across participants for the three tracking protocols. Boxplots on the right show reduced variability for the unimodal Xtract protocol. Both the unimodal (Xtract) and combined (Ctract) protocols outperform the multimodal Ftract approach. Significant *t*-tests are indicated by lines: dotted for *p* < 0.05, dashed for *p* < 0.01, and solid for *p* < 0.001 (Holm-Bonferroni correction)

Beyond tract-map similarity, tract quality was further assessed using normalized streamline counts, a connectivity measure, which we compared across protocols. Bilateral FAT, AF, and UF showed the highest values with the Xtract protocol and the lowest with Ftract, while Ctract yielding intermediate values (Fig. 6). In contrast, for the bilateral SLF3, ILF, and the right MdLF, normalized streamline counts were highest with Ctract, followed by the Xtract, and lowest with Ftract. An exception was observed for the left MdLF, which showed the lowest values under Xtract (Fig. 6). Full test statistics are reported in Supplementary Table 6.

## Discussion

The aim of this study was to evaluate the spatial similarity and comparability of three tractography protocols for reconstructing language-related white matter tracts. Across all cross-correlation analyses, Xtract-Ctract alignment was consistently stronger than Xtract-Ftract. Both Xtract and Ctract alignments were marginally lower than, but remained consistent with, benchmark values reported for the 1000-subject HCP cohort [8]. Normalized streamline counts further revealed the highest connectivity values for Xtract and Ctract, whereas the purely fMRI-guided approach showed the lowest normalized streamline counts, indicating weaker structural connectivity and, therefore, a potentially reduced ability to capture the underlying white matter pathways relevant for language function. We acknowledge that normalized streamline counts represent only a proxy measure of structural connectivity, and can be affected by tract length, curvature, and the presence of crossing fibers, which may introduce bias when comparing tracts with distinct anatomical characteristics [68]. However, the present study focused on within-tract comparisons to evaluate differences between tracking protocols rather than across-tract variations. Thus, these anatomical factors are unlikely to confound our main findings.

These findings indicate that Xtract provides a robust framework for reconstructing frontal and dorsal language-related white matter tracts. In contrast, fMRI alone appears insufficient for reliable tract reconstruction and even failed to reconstruct 17 tracts (Supplementary Table 8), likely because fMRI-defined seeds are prone to individual variability [69, 70], task dependency [71, 72], and signal noise [73]. By incorporating standardized anatomical priors [74, 75], as implemented in Xtract and Ctract, tractography is constrained to anatomically plausible subcortical pathways. Consistent with previous work on other major bundles such as the optic radiation [76], our findings highlight the important role of anatomical priors in achieving robust and reliable tract reconstruction.

Beyond these general findings, our study also highlights tract-specific differences. Ventral tracts such as the MdLF and ILF benefited from a multimodal approach combining fMRI guidance with Xtract masks. One possible methodological explanation is that, within Xtract, these tracts are reconstructed using reverse tracking [8], a strategy not applied to the frontal and dorsal tracts or the uncinate fasciculus, for which Xtract alone outperformed Ctract. The fact that the ILF and the MdLF protocols already required optimization by the Xtract developers underscores their complexity and variability. Thus, while subcortical anatomical priors provide a strong foundation for most pathways, tracts with more variable anatomy and higher inter-subject heterogeneity such as ILF and MdLF [77, 78] may require complementary functional information. These findings emphasize the importance of tailoring tracking strategies to tract-specific characteristics, with multimodal approaches offering particular advantages for ventral white matter tracts.

Although Warrington and colleagues [8] based their analyses on large population cohorts (HCP and UK Biobank), the analytical framework they proposed, that is assessing within- and across-cohort similarity as indicators of tractography consistency, can also be applied to smaller samples. In the present study, this approach served not to replicate population-level statistics, but to evaluate whether our independently acquired dataset yields qualitatively comparable patterns of tract overlap when analyzed under the same methodological conditions. While smaller sample sizes naturally limit statistical power and generalizability, such comparisons remain informative for situating our findings within an established reference framework. Furthermore, it should be noted that similarity metrics in Warrington et al. were strongly influenced by genetic and demographic factors, as evidenced by higher correlations within cohorts and among monozygotic twins. Given that our cohort represents a different genetic background, somewhat lower similarity values are expected and do not necessarily reflect reduced methodological robustness.

The biological accuracy of fiber tractograms has improved, yet it is important to recognize that even state-of-the-art techniques still face unresolved issues that introduce substantial bias into structural connectomes [79]. A key point when interpreting probabilistic fiber tracking maps generated with FSL’s *probtrackx2* (and thus Xtract) is that they do not quantify the true underlying anatomy, but rather the consistency with which streamlines are repeatedly sampled [79]. In addition, reliable tractography near the cortex remains difficult due to modeling errors and geometric biases [80], which poses a major limitation for fMRI-guided tracking where seed information lies in cortical gray matter. Ultimately, validation against ground truth remains notoriously challenging, and appropriate techniques are still under development [81, 82]. Taken together, diffusion tractography can only complement, but not replace, intraoperative direct electrical stimulation for mapping functional pathways during brain surgery.

A limitation of the present study concerns the comparison with the HCP benchmark reported by Warrington and colleagues [8]. While the underlying HCP diffusion data are openly accessible, the tract-specific similarity metrics from that study are not available. As a result, our comparison was based on the published summary statistics for all 46 Xtract tracts, rather than on tract-specific values for the 12 language-related pathways examined here. Future work could extend this analysis by recomputing tract-specific benchmarks directly from the HCP dataset to allow a more precise comparison.

A further limitation is the relatively small sample size. Although adequate for the methodological comparisons conducted here, larger cohorts will be needed to confirm the generalizability of the findings. Moreover, while the motivation for this study arose from the clinical context of preoperative mapping of language-related brain structures, the present sample consisted of neurotypical young adults. As such, the findings cannot be generalized beyond language tracts or to patient populations. Future work will be needed to evaluate the two superior tracking protocols in clinical cohorts.

## Conclusion

Automated, anatomically informed tractography (e.g., Xtract, Ctract) yields more similar reconstructions of language-related white matter pathways in neurotypical subjects than purely fMRI-guided tracking, with particular advantages for ventral and frontal pathways. Ventral tracts such as the ILF and MdLF appear to benefit from multimodal approaches that combine subcortical anatomical priors with fMRI guidance. Overall, anatomically informed protocols offer a strong foundation for tractography in both research and clinical contexts, while individualized hybrid strategies may be particularly valuable for neurosurgical planning. Future work should further refine these approaches and evaluate their applicability across patient populations and additional functional networks.

## Supplementary Information

ESM: Supplementary material 1

## Abbreviations

AF: arcuate fasciculus
AG: angular gyrus
aSMG: anterior division of the supramarginal gyrus
AntTemp: anterior temporal region
Ctract: combined XTRACT and functional-MRI guided tracking protocol
dMRI: diffusion MRI
EPI: echo planar imaging
FAT: frontal aslant tract
fMRI: functional MRI
Ftract: functional-MRI guided tracking protocol
IFG: inferior frontal gyrus
IFL: inferior longitudinal fasciculus
IFGop: Inferior frontal gyrus pars opercularis
IFGorb: inferior frontal gyrus pars orbitalis
MdLF: middle longitudinal fasciculus
NSI: normalized streamline index
pCG: precentral gyrus
pSMG: posterior division of the supramarginal gyrus
PostTemp: posterior temporal region
SFG: superior frontal gyrus
STG: superior temporal gyrus
ROI: region of interest
SLF3: superior longitudinal fasciculus III
SMA: supplementary motor area
TP: temporal pole
UF: uncinate fasciculus
Xtract: XTRACT protocol
