# Supplementary material for: Evaluating Unimodal and Multimodal Tracking Strategies for the Reconstruction of Language-related White Matter Tracts
Source: Clin Neuroradiol. 2025 Dec 10;36(2):485–97. doi: 10.1007/s00062-025-01601-9 (PMC13320052; doi:10.1007/s00062-025-01601-9)
Supplement: Supplementary file 1 — ESM: Supplementary material 1 [file 62_2025_1601_MOESM1_ESM.docx]

**Evaluating unimodal and multimodal tracking strategies for the reconstruction of language-related white matter tracts**

**Supplementary Table 1** MNI coordinates of cluster maxima and local maxima derived from a mixed-effects analysis across all participants for the contrast compound > pseudoword rhyme (*Z* ≥ 3.7, *p* < 0.05).

| Region | Hemisphere | Z-score | X | Y | Z | Voxels |
| --- | --- | --- | --- | --- | --- | --- |
| Cerebellum VIIb | R | 6.27 | 38 | -64 | -54 | 11570 |
| Cerebellum VI | L | 6.11 | -30 | -60 | -24 |  |
| Inferior temporal gyrus, posterior division | L | 6.06 | -44 | -32 | -18 |  |
| Temporal fusiform cortex, posterior division | L | 6 | -38 | -30 | -24 |  |
| Cerebellum VI | R | 6 | 28 | -58 | -28 |  |
| Cerebellum Crus II | R | 5.98 | 14 | -80 | -48 |  |
| Middel Temporal gyrus | L | 5.53 | -48 | -52 | 0 |  |
| Frontal orbital Cortex | L | 6.42 | -30 | 32 | -2 | 8335 |
| Inferior frontal gyrus pars opercularis | L | 6.36 | -50 | 20 | 18 |  |
| Paracingulate gyrus | L | 6.34 | -8 | 18 | 40 |  |
| Frontal operculum cortex | L | 6.16 | -38 | 30 | 6 |  |
| Orbital cortex | L | 5.98 | -38 | 28 | -2 |  |
| Inferior frontal gyrus pars triangularis | L | 5.89 | -44 | 24 | 20 |  |
| Insular Cortex | R | 5.9 | 36 | 24 | -2 | 1407 |
| Frontal orbital cortex | R | 5.69 | 38 | 30 | -8 |  |
| Insular cortex | R | 5.51 | 40 | 18 | -6 |  |
| Insular cortex | R | 5.35 | 28 | 20 | 12 |  |
| Frontal operculum cortex | R | 5.1 | 32 | 22 | 14 |  |
| Temporal pole | R | 4.89 | 50 | 14 | -18 |  |
| Anterior intra-parietal sulcus hIP3 | L | 4.97 | -28 | -70 | 40 | 703 |
| Lateral occipital cortex, superior division | L | 4.73 | -28 | -60 | 34 |  |
| Lateral occipital cortex, superior division | L | 4.7 | -26 | -62 | 40 |  |
| Superior parietal lobule | L | 4.67 | -26 | -46 | 46 |  |
| Lateral occipital cortex, superior division | L | 4.61 | -22 | -74 | 34 |  |
| Lateral occipital cortex, superior division | L | 4.32 | -32 | -62 | 50 |  |
| Precentral gyrus | R | 4.32 | 30 | -8 | 54 | 182 |
| Middel frontal gyrus | R | 4.12 | 34 | -2 | 58 |  |
| Precentral gyrus | R | 4.07 | 34 | 0 | 32 |  |
| Precentral gyrus | R | 3.98 | 30 | -4 | 40 |  |
| Pallidum | R | 4.96 | 14 | 0 | -4 | 136 |
| Cerebellum VIIIa | L | 4.53 | -28 | -62 | -54 | 120 |
| Cerebellum VIIb | L | 4.43 | -38 | -60 | -54 |  |
| Cerebellum VIIIa | L | 4.02 | -22 | -62 | -46 |  |
| Putamen | R | 4.47 | 20 | 0 | 10 | 73 |
| Anterior intra-parietal sulcus hIP1 | L | 4.34 | -28 | -48 | 30 | 57 |
| Brainstem |  | 4.38 | 0 | -40 | -44 | 55 |
| Anterior intra-parietal sulcus hIP1 | R | 4.43 | 32 | -60 | 34 | 54 |
| Lateral occipital cortex, superior division | R | 4 | 24 | -56 | 36 |  |
| Supramarginal gyrus posterior division | R | 4.3 | 30 | -40 | 36 | 54 |

**Supplementary Table 2** MNI coordinates of cluster maxima and local maxima derived from a mixed-effects analysis across all participants for the contrast pseudoword rhyme > compound (Z ≥ 3.7, p < 0.05).

| Region | Hemisphere | Z-score | X | Y | Z | Voxels |
| --- | --- | --- | --- | --- | --- | --- |
| Frontal pole | R | 5.92 | 2 | 76 | 4 | 5000 |
| Frontal pole |  | 5.7 | 0 | 72 | 12 |  |
| Frontal medial cortex |  | 5.69 | 0 | 48 | -12 |  |
| Cingulate gyrus, anterior division |  | 5.64 | 0 | 40 | -6 |  |
| Frontal pole | R | 5.43 | 14 | 66 | 18 |  |
| Superior frontal gyrus | R | 5.39 | 14 | 34 | 54 |  |
| Precuneous cortex | L | 6.12 | -4 | -64 | 32 | 4387 |
| Precuneous cortex | R | 5.92 | 16 | -58 | 14 |  |
| Cingulate gyrus | L | 5.81 | -4 | -26 | 40 |  |
| Cingulate gyrus, posterior division | R | 5.74 | 4 | -40 | 40 |  |
| Cingulate gyrus, posterior division | L | 5.71 | -4 | -34 | 44 |  |
| Precuneous cortex | L | 5.67 | -4 | -70 | 28 |  |
| Lateral occipital cortex, superior division | R | 5.55 | 50 | -62 | 30 | 1115 |
| Lateral occipital cortex, superior division | R | 5.5 | 46 | -72 | 38 |  |
| Lateral occipital cortex, superior division | R | 5.47 | 46 | -64 | 30 |  |
| Lateral occipital cortex, superior division | R | 5.24 | 48 | -66 | 36 |  |
| Lateral occipital cortex, superior division | R | 5.23 | 48 | -70 | 44 |  |
| Supramarginal gyrus, posterior division | R | 4.01 | 58 | -46 | 42 |  |
| Middle temporal gyrus, posterior division | R | 4.93 | 64 | -12 | -18 | 828 |
| Middle temporal gyrus, anterior division | R | 4.87 | 58 | -2 | -34 |  |
| Middle temporal gyrus, posterior division | R | 4.73 | 72 | -16 | -18 |  |
| Temporal pole | R | 4.61 | 48 | 6 | -50 |  |
| Inferior temporal gyrus, anterior division | R | 4.47 | 48 | 4 | -46 |  |
| Middle temporal gyrus, posterior division | R | 4.4 | 62 | -12 | -28 |  |
| Cerebellum Crus I | L | 4.98 | -48 | -68 | -42 | 745 |
| Cerebellum Crus II | L | 4.93 | -40 | -70 | -46 |  |
| Cerebellum Crus I | L | 4.74 | -30 | -80 | -36 |  |
| Cerebellum Crus II | L | 4.69 | -16 | -86 | -42 |  |
| Cerebellum Crus II | L | 4.63 | -36 | -82 | -48 |  |
| Inferior parietal lobule | L | 4.86 | -48 | -60 | 48 | 506 |
| Angular gyrus | L | 4.74 | -44 | -58 | 44 |  |
| Lateral occipital cortex, superior division | L | 4.7 | -50 | -60 | 54 |  |
| Lateral occipital cortex, superior division | L | 4.41 | -42 | -76 | 36 |  |
| Lateral occipital cortex, superior division | L | 4.38 | -40 | -72 | 36 |  |
| Angular gyrus | L | 4.02 | -54 | -60 | 28 |  |
| Frontal pole | L | 4.81 | -20 | 52 | 40 | 417 |
| Middle frontal gyrus | L | 4.44 | -28 | 30 | 38 |  |
| Middle frontal gyrus | L | 4.32 | -34 | 32 | 48 |  |
| Middle frontal gyrus | L | 4.3 | -32 | 36 | 42 |  |
| Frontal pole | L | 4.3 | -20 | 58 | 34 |  |
| Middle frontal gyrus | L | 4.18 | -40 | 20 | 44 |  |
| Planum polare | R | 5.35 | 54 | -4 | 0 | 198 |
| Heschel’s gyrus/ Insular cortex | R | 4.42 | 38 | -24 | 6 |  |
| Planum polare | R | 4.26 | 40 | -20 | -2 |  |
| Heschel’s gyrus/ Central opercular | R | 4.24 | 48 | -10 | 4 |  |
| Planum polare | R | 4.23 | 44 | -10 | -10 |  |
| Insular cortex / Planum polare | R | 4.19 | 44 | -6 | -6 |  |
| Frontal pole | L | 4.99 | -28 | 66 | -4 | 196 |
| Frontal pole | L | 4.76 | -22 | 66 | 6 |  |
| Middle Teporal gyrus, posterior division | L | 4.8 | -68 | -22 | -14 | 108 |
| Middle temporal gyrus, posterior division | L | 4.65 | -70 | -24 | -20 |  |
| Middle temporal gyrus, posterior division | L | 4.22 | -66 | -16 | -26 |  |
| Subcallosal cortex |  | 5.23 | 0 | 12 | -14 | 100 |
| Cerebellum Crus II | R | 5.4 | 46 | -52 | -44 | 92 |
| Cerebellum Crus I | R | 3.85 | 48 | -60 | -42 |  |
| Superior frontal gyrus | L | 4.84 | -20 | 18 | 56 | 89 |
| Superior frontal gyrus | L | 4.1 | -14 | 20 | 62 |  |
| Precuneous cortex | R | 4.2 | 2 | -54 | 70 | 69 |
| Precuneous cortex | R | 4.12 | -4 | -52 | 70 |  |
| Postcentral gyrus | R | 3.96 | -4 | -42 | 76 |  |
| Postcentral gyrus | R | 3.94 | -2 | -42 | 70 |  |
| Cerebellum IX | L | 5.7 | -4 | -54 | -52 | 50 |
| Middle temporal gyrus, posterior division | L | 4.26 | -62 | -6 | -34 | 47 |
| Middle temporal gyrus, anterior division | L | 4.24 | -60 | -2 | -30 |  |

**Supplementary Table 3** Within-subject similarity values (Fishers *Z*-transformed *r*)

| **Tract** | **Ctract-Xtract** | **One-sample *t*-test** | | **Ftract-Xtract** | **One sample *t*-test** | | **paired *t*-test**  **Xtract-Ctract vs Xtract-Ftract** | |
| --- | --- | --- | --- | --- | --- | --- | --- | --- |
|  | Mean  [95% CI] | *t*(*df*) | *p* | Mean  [95% CI] | *t*(*df*) | *p* | *t*(*df*) | *p* |
| *left* |  |  |  |  |  |  |  |  |
| **FAT** | 1.32  [1.24-1.40] | 5.95 (19) | 0.01002 | 0.74  [0.66-0.80] | -10.67 (19) | 1.85e-09 | 29.40 (19) | 2.63e-17 |
| **AF** | 1.28  1.24-1.40 | 4.08 (19) | 0.0006412 | 0.73  [0.67-0.80] | -11.68 (19) | 4.074e-10 | 11.07 (19) | 9.99e-10 |
| **SLF3** | 0.78  [1.24-1.40] | -8.20 (19) | 0.0001158 | 0.27  [0.23-0.32] | -37.39 (19) | < 2.2e-16 | 11.89 (19) | 3.01e-10 |
| **ILF** | 0.83  [0.72-0.94] | -5.03 (19) | 0.07507 | 0.34  [0.28-0.40] | -27.69 (19) | < 2.2e-16 | 6.58 (19) | 2.68e-06 |
| **MdLF** | 0.87  [0.80-0.94] | -6.72 (19) | 0.002007 | 0.42  [0.36-0.48] | -22.14 (18) | 1.655e-14 | 10.11 (18) | 7.52e-09 |
| **UF** | 1.34  [1.12-1.55] | 2.34 (18) | 0.03076 | 0.35  [0.29-0.42] | -24.13 (17) | 1.369e-14 | 10.28 (17) | 1.02e-08 |
| *right* |  |  |  |  |  |  |  |  |
| **FAT** | 1.00  [0.88-1.13] | -1.65 (18) | 0.1163 | 0.49  [0.38-0.61] | -11.18 (16) | 5.714e-09 | 27.26 (16) | 7.72e-15 |
| **AF** | 1.56  [1.32-1.78] | 4.11 (17) | 0.0007251 | 0.40  [0.31-0.49] | -16.79 (15) | 3.912e-11 | 8.23 (15) | 6.03e-07 |
| **SLF3** | 0.63  [0.55-0.71] | -12.16 (19) | 2.084e-10 | 0.29  [0.23-0.35] | -27.51 (18) | 3.696e-16 | 7.93 (18) | 1.301e-08 |
| **ILF** | 1.73  [1.5-1.96] | 5.70 (18) | 2.089e-05 | 0.30  [0.22-0.38] | -22.29 (17) | 5.073e-14 | 10.40 (16) | 1.59e-08 |
| **MdLF** | 0.84  [0.80-0.89] | -12.13 (19) | 2.169e-10 | 0.38  [0.32-0.44] | -26.19 (19) | 2.261e-16 | 16.09 (19) | 1.59e-12 |
| **UF** | 1.62  [1.44-1.80] | 6.01 (18) | 1.105e-05 | 0.22  [0.17-0.27] | -36.58 (18) | < 2.2e-16 | 14.44 (18) | 2.42e-11 |

**Supplementary Table 4** Intra-cohort similarity values (Fishers *Z*-transformed *r*)

| **Tract** | **Ctract-Xtract** | **One-sample *t*-test** | | **Ftract-Xtract** | **One-sample *t-*test** | | **paired *t*-test**  **Xtract-Ctract vs Xtract-Ftract** | |
| --- | --- | --- | --- | --- | --- | --- | --- | --- |
|  | Mean  [95% CI] | *t*(*df*) | *p* | Mean  [95% CI] | *t*(*df*) | *p* | *t*(*df*) | *p* |
| *left* |  |  |  |  |  |  |  |  |
| **FAT** | 0.60  [0.59-0.62] | 6.65 (19) | 2.336e-06 | 0.51  [0.49-0.52] | -7.34 (19) | 5.835e-07 | 19.03 (19) | 7.82e-14 |
| **AF** | 0.51  [0.50-0.53] | -6.19 (19) | 6.012e-06 | 0.42  [0.41-0.44] | -17.22 (19) | 4.744e-13 | 30.60 (19) | 1.25e-17 |
| **SLF3** | 0.45  [0.43-0.46] | -14.57 (19) | 9.135e-12 | 0.24  [0.23-0.25] | -70.84 (19) | < 2.2e-16 | 41.53 (19) | 4.11e-20 |
| **ILF** | 0.46  [0.45-0.47] | -22.58 (19) | 3.461e-15 | 0.24  [0.23-0.25] | -58.19 (19) | < 2.2e-16 | 39.58 (19) | 1.01e-19 |
| **MdLF** | 0.49  [0.49-0.50] | -17.33 (19) | 4.258e-13 | 0.31  [0.30-0.31] | -70.36 (19) | < 2.2e-16 | 100.03 (19) | 2.49e-27 |
| **UF** | 0.55  [0.53-0.56] | -2.01 (19) | 0.05887 | 0.27  [0.25-0.28] | -41.56 (19) | < 2.2e-16 | 33.03 (19) | 3.01e-18 |
| *right* |  |  |  |  |  |  |  |  |
| **FAT** | 0.51  [0.49-0.52] | -7.62 (19) | 3.429e-07 | 0.34  [0.32-0.35] | -34.44 (19) | < 2.2e-16 | 34.23 (19) | 1.54e-18 |
| **AF** | 0.50  [0.48-0.51] | -8.72 (19) | 4.578e-08 | 0.27  [0.26-0.28] | -59.13 (19) | < 2.2e-16 | 49.89 (19) | 1.30e-21 |
| **SLF3** | 0.34  [0.32-0.35] | -31.27 (19) | < 2.2e-16 | 0.23  [0.22-0.24] | -67.25 (19) | < 2.2e-16 | 22.69 (19) | 3.17e-15 |
| **ILF** | 0.57 [0.55-0.59] | 1.15 (19) | 0.2655 | 0.21 [0.20-0.22] | -66.42 (19) | < 2.2e-16 | 47.01 (19) | 3.98e-21 |
| **MdLF** | 0.52 [0.51-0.53] | -12.84 (19) | 8.25e-11 | 0.30 [0.29-0.31] | -92.12 (19) | < 2.2e-16 | 67.83 (19) | 3.91e-24 |
| **UF** | 0.62 [0.61-0.64] | 9.01 (19) | 2.727e-08 | 0.18 [0.17-0.19] | -76.97 (19) | < 2.2e-16 | 78.88 (19) | 2.24e-25 |

**Supplementary Table 5** Across cohort similarity values (Fishers *Z*-transformed *r*)

|  | **Left** | **One-sample *t*-test** | | **Right** | **One-sample *t*-Test** | |
| --- | --- | --- | --- | --- | --- | --- |
|  | Mean  [95% CI] | *t* (*df*) | *p* | Mean  [95% CI] | *t* (*df*) | *p* |
| **Xtract** | 0.38  [0.38-0.39] | -10.26 (19) | 3.501e-09 | 0.36  [0.36-0.37] | -14.38 (19) | 1.152e-11 |
| **Ctract** | 0.38  [0.37-0.39] | -11.22 (19) | 7.976e-10 | 0.36  [0.34-0.37] | -19.09 (19) | 7.407e-14 |
| **Ftract** | 0.25  [0.23-0.27] | -25.42 (19) | 3.904e-16 | 0.21  [0.19-0.23] | -21.86 (19) | 6.294e-15 |

**Supplementary Table 6** Normalized streamline counts

| **Tract** | **Xtract** | **Ctract** | **Ftract** | **Ctract-Xtract** | | **Ftract-Xtract** | | **Ctract-Ftract** | |
| --- | --- | --- | --- | --- | --- | --- | --- | --- | --- |
|  | Mean [95% CI] | Mean [95% CI] | Mean [95% CI] | *t*(*df*) | *p* | *t*(*df*) | *p* | *t*(*df*) | *p* |
| *left* |  |  |  |  |  |  |  |  |  |
| **FAT** | 0.77  [0.75-0.78] | 0.72  [0.70-0.74] | 0.64  [0.61-0.69] | -7.19 (19) | 2.36e-06 | -6.48 (19) | 6.58e-06 | 4.98 (19) | 8.24e-05 |
| **AF** | 0.80  [0.79-0.82] | 0.72  [0.71-0.73] | 0.63  [0.61-0.65] | -25.17 (19) | 1.41e-15 | -17.50 (19) | 7.10e-13 | 9.67 (19) | 9.05e-09 |
| **SLF3** | 0.64  [0.611-0.66] | 0.80  [0.78-0.81] | 0.52  [0.47-0.58] | 12.69 (19) | 3.00e-10 | -3.89 (18) | 1.00e-03 | 12.87 (18) | 3.24e-10 |
| **ILF** | 0.78  [0.77-0.80] | 0.84  [0.82-0.86] | 0.72  [0.68-0.76] | 6.65 (19) | 4.68e-06 | -3.20 (19) | 0.005 | 7.31 (19) | 1.85e-06 |
| **MdLF** | 0.73  [0.72-0.73] | 0.85  [0.84-0.87] | 0.76  [0.72-0.80] | 24.5 (19) | 2.32e-15 | 1.64 (18) | 0.118 | 4.92 (18) | 0.00022 |
| **UF** | 0.80  [0.78-0.82] | 0.75  [0.73-0.77] | 0.48  [0.41-0.54] | -4.89 (19) | 1.02e-04 | -10.41 (17) | 1.72e-08 | 10.80 (17) | 1.49e-08 |
| *rigth* |  |  |  |  |  |  |  |  |  |
| **FAT** | 0.75  [0.73-0.76] | 0.59  [0.54-0.65] | 0.45  [0.36-0.54] | -6.790 (18) | 3.08e-06 | -7.38 (16) | 3.08e-06 | 7.64 (16) | 3.00e-06 |
| **AF** | 0.72  [0.70-0.74] | 0.65  [0.63-0.67] | 0.40  [0.32-0.48] | -22.58 (19) | 1.04e-14 | -8.76 (15) | 5.50e-07 | 6.82 (15) | 5.79e-06 |
| **SLF3** | 0.65  [0.64-0.67] | 0.80  [0.78-0.81] | 0.59  [0.55-0.62] | 12.16 (19) | 6.24e-10 | -3.44 (17) | 3.00e-03 | 11.88 (17) | 2.36e-09 |
| **ILF** | 0.79  0.78-0.80 | 0.81  [0.80-0.82] | 0.55  [0.45-0.64] | 3.81 (19) | 0.001 | -5,41 (17) | 9.32e-05 | 5.95 (17) | 4.74e-05 |
| **MdLF** | 0.74  [0.73-0.75] | 0.85  [0.84-0.85 | 0.63  [0.55-0.71] | 33.10 (19) | 8.67e-18 | -3,02 (19) | 0.007 | 5.92 (19) | 2.14e-05 |
| **UF** | 0.82  [0.81-0.84] | 0.74  [0.73-0.75] | 0.39  [0.29-0.50] | -17.96 (19) | 6.72e-13 | -8.87 (17) | 1.75e-07 | 7.37 (17) | 1.09e-06 |

**Supplementary Table 7** Anatomical constraints for fMRI-guided protocols

| **Tract** | **Seed** | **Target** | **Overlap-Map**  **left hemisphere** | **Overlap-Map**  **right hemisphere** |
| --- | --- | --- | --- | --- |
| FAT | SFG**  SMA** | IFG* | 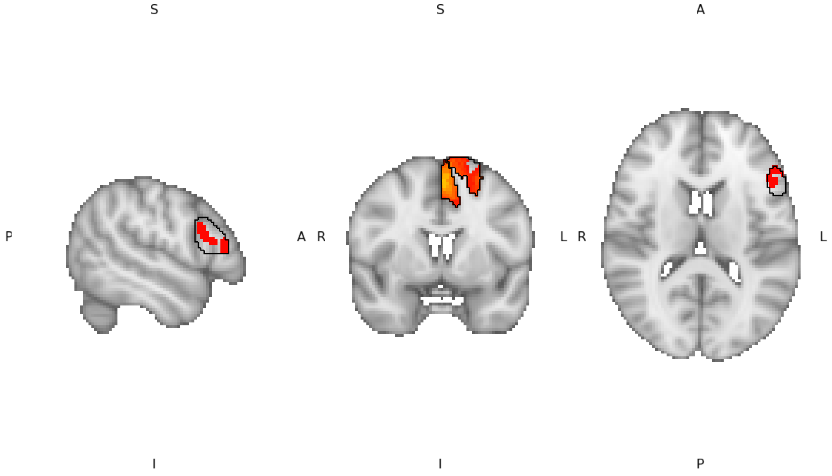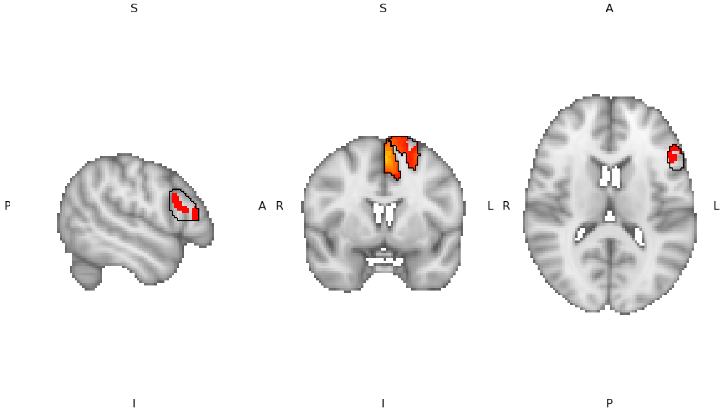 | 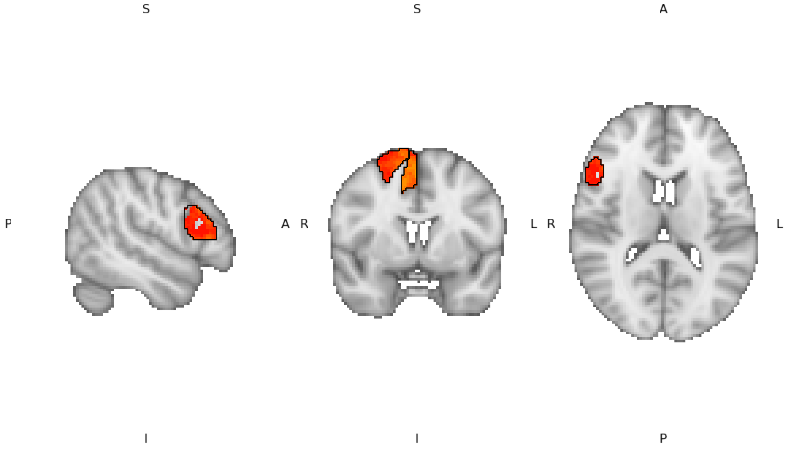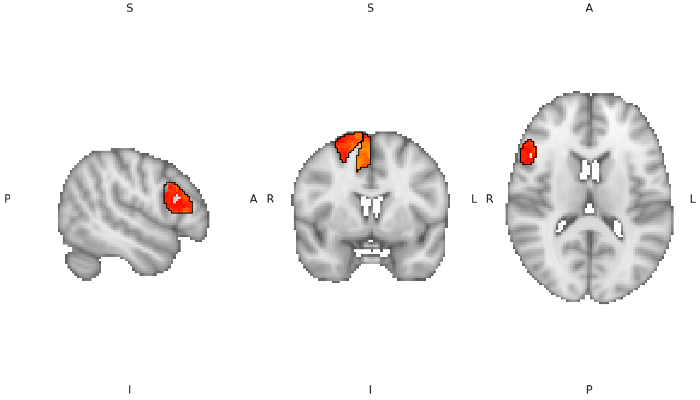 |
| AF | IFG* | PostTemp* | 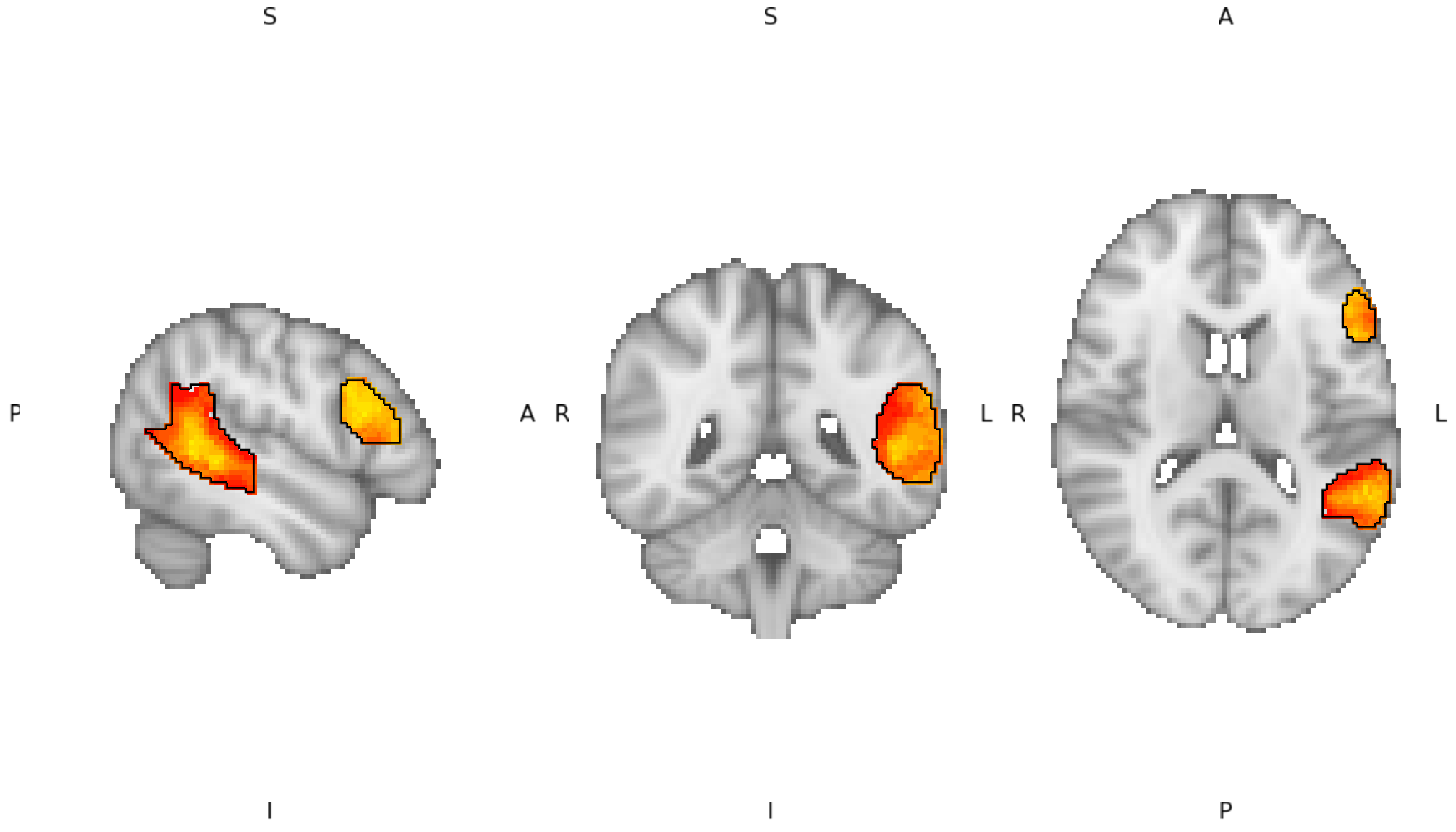 | 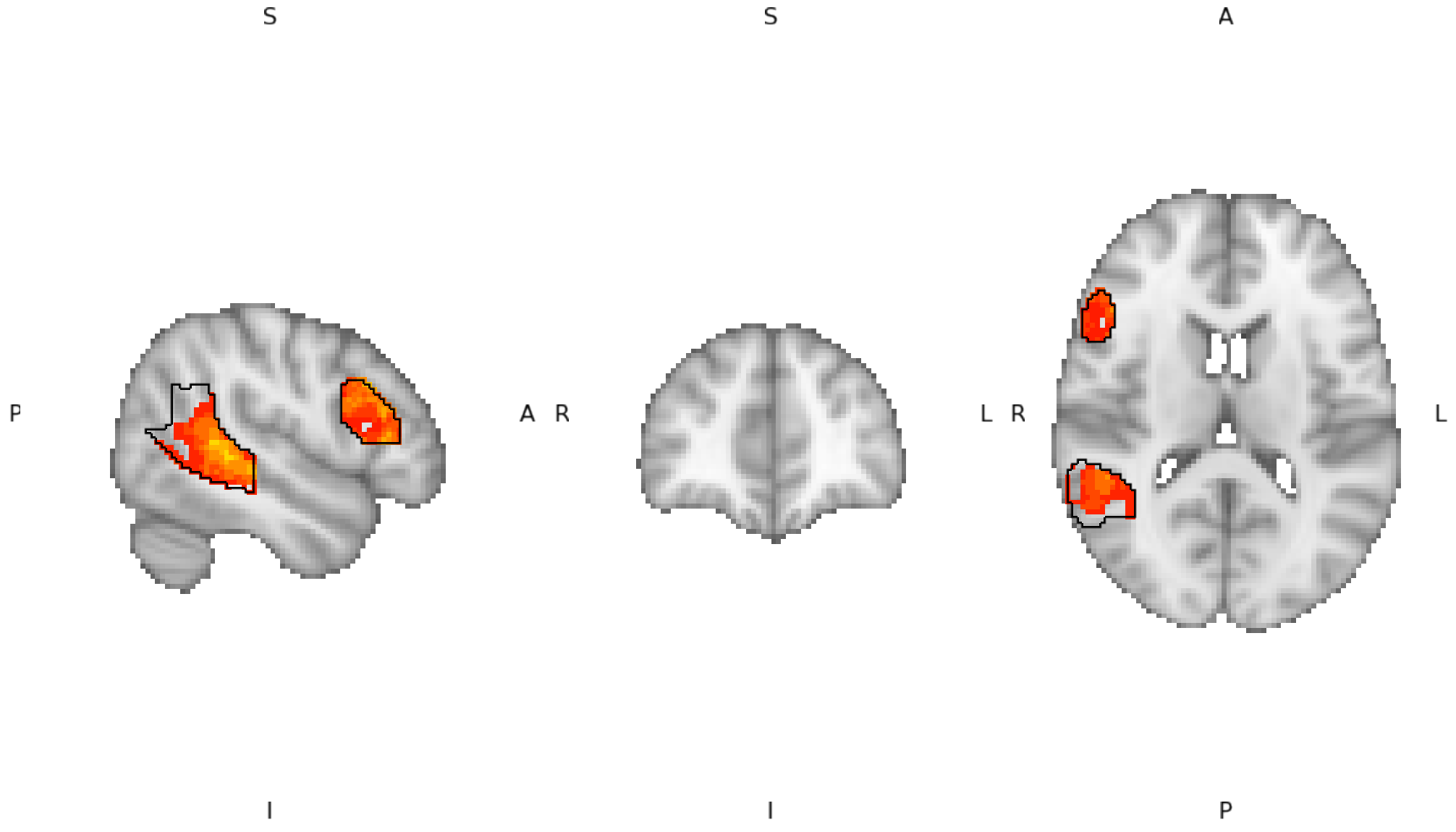 |
| SLF3 | pCG**  IFGop** | aSMG**  pSMG** | 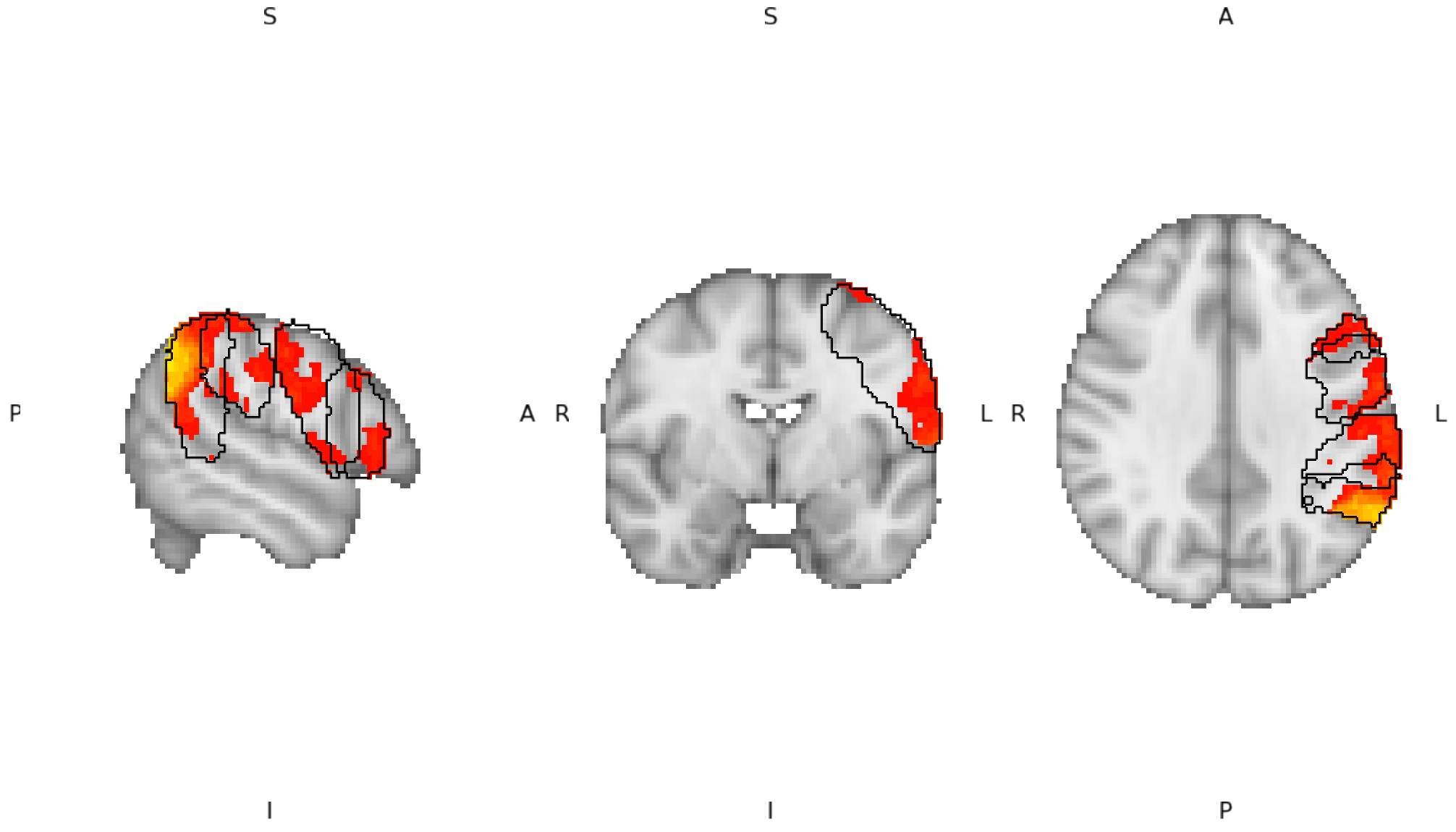 | 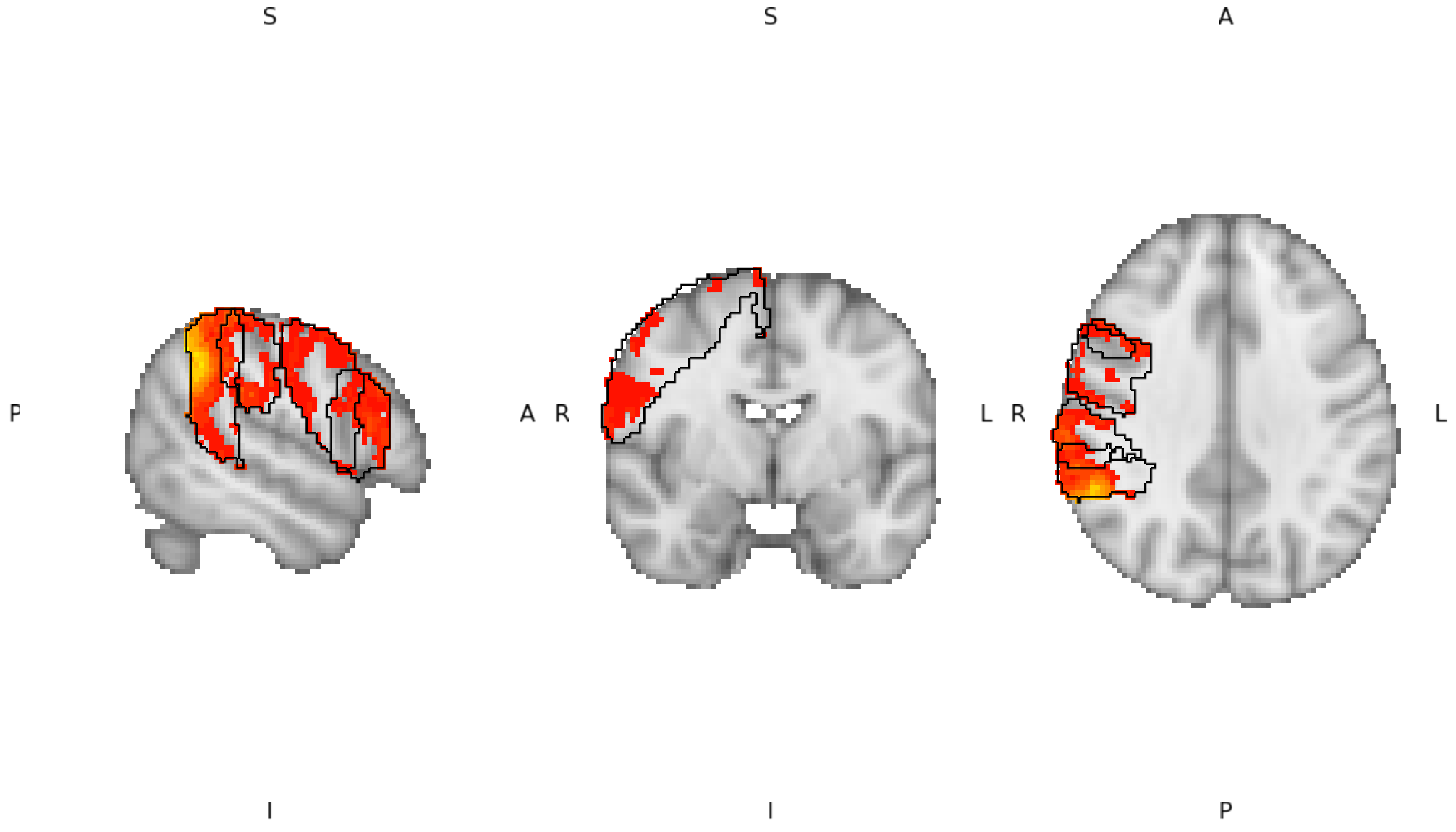 |
| ILF | PostTemp* | AntTemp* | 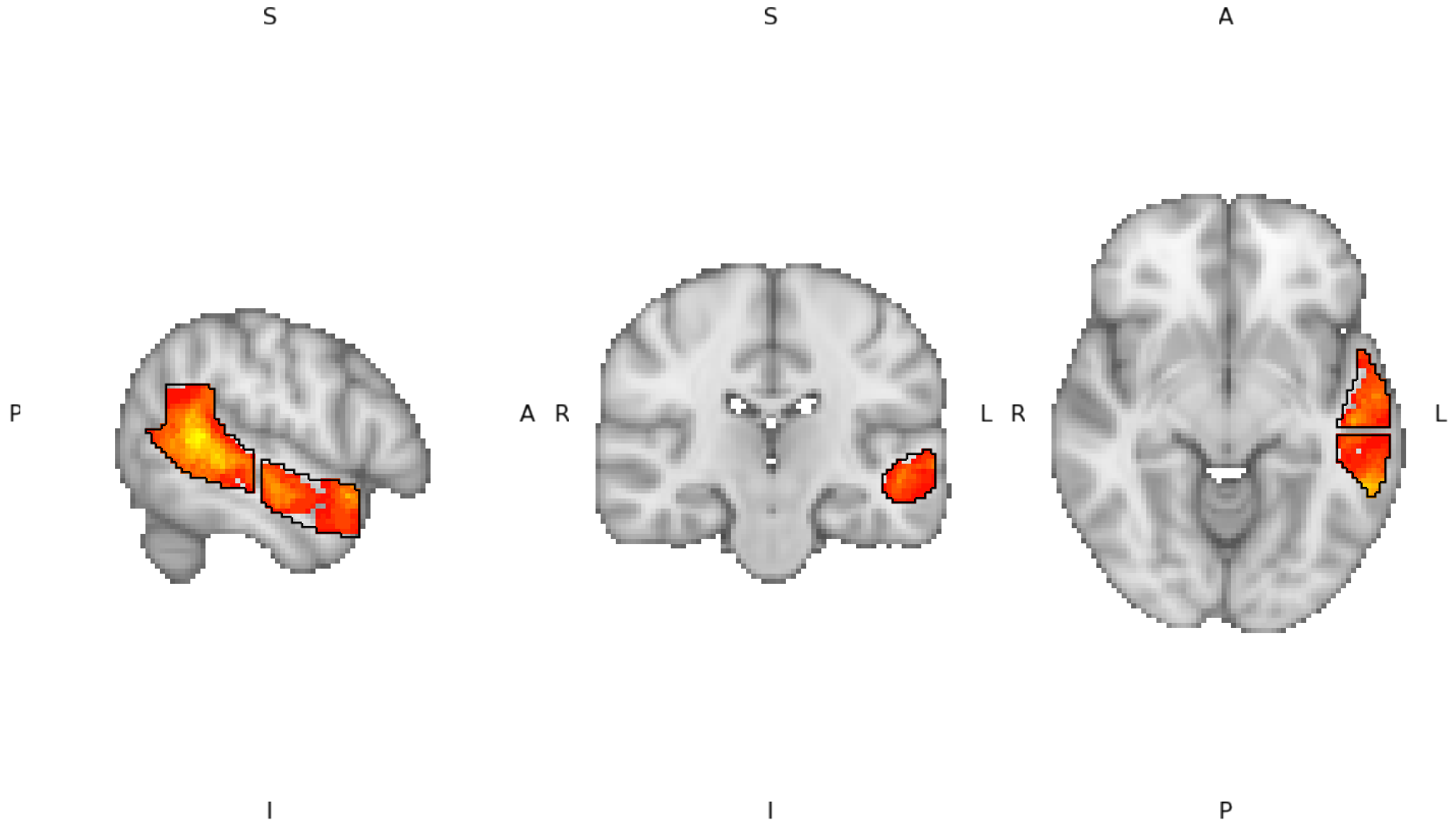 | 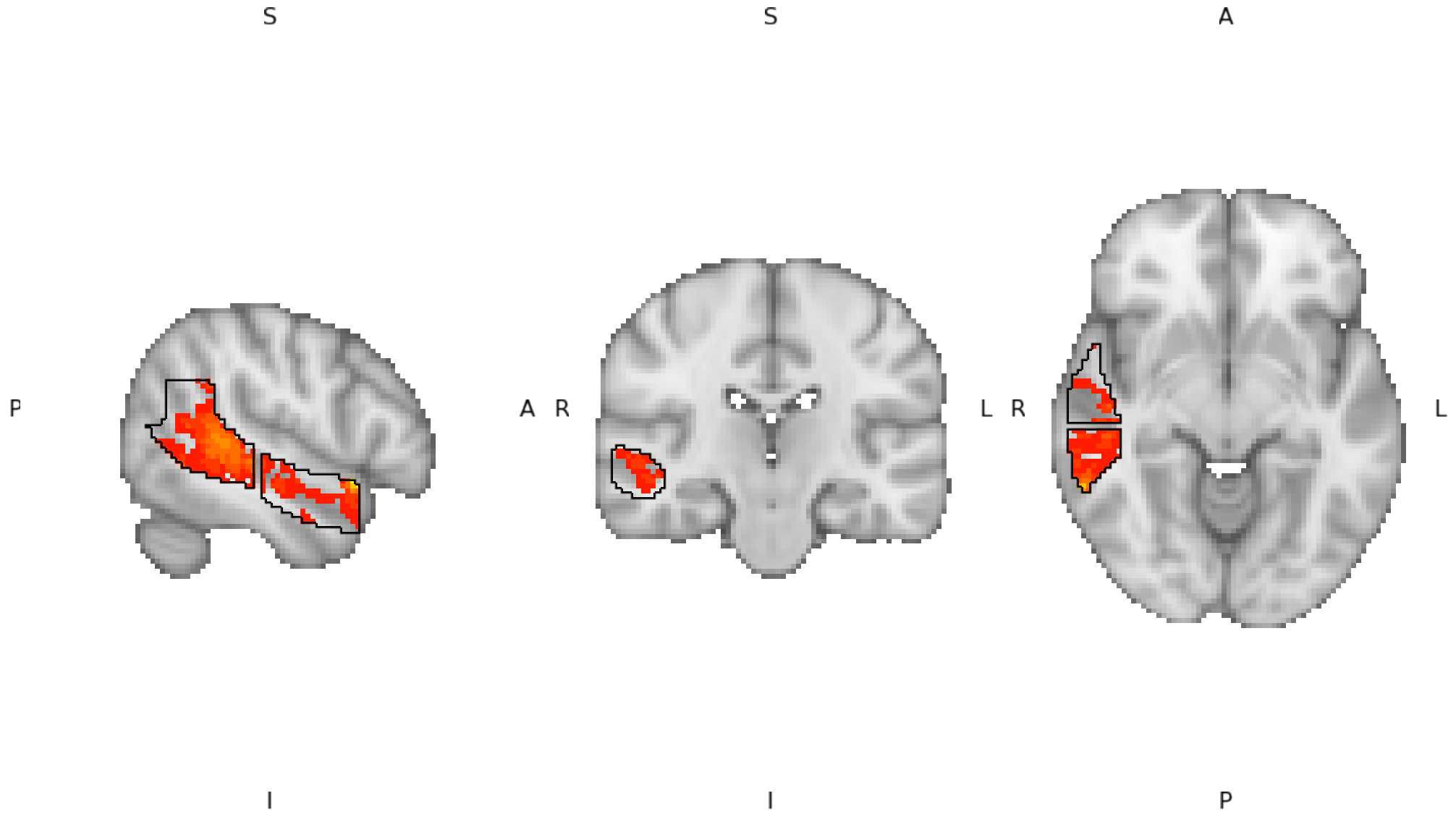 |
| UF | AntTemp* | IFG*  IFGorb* | 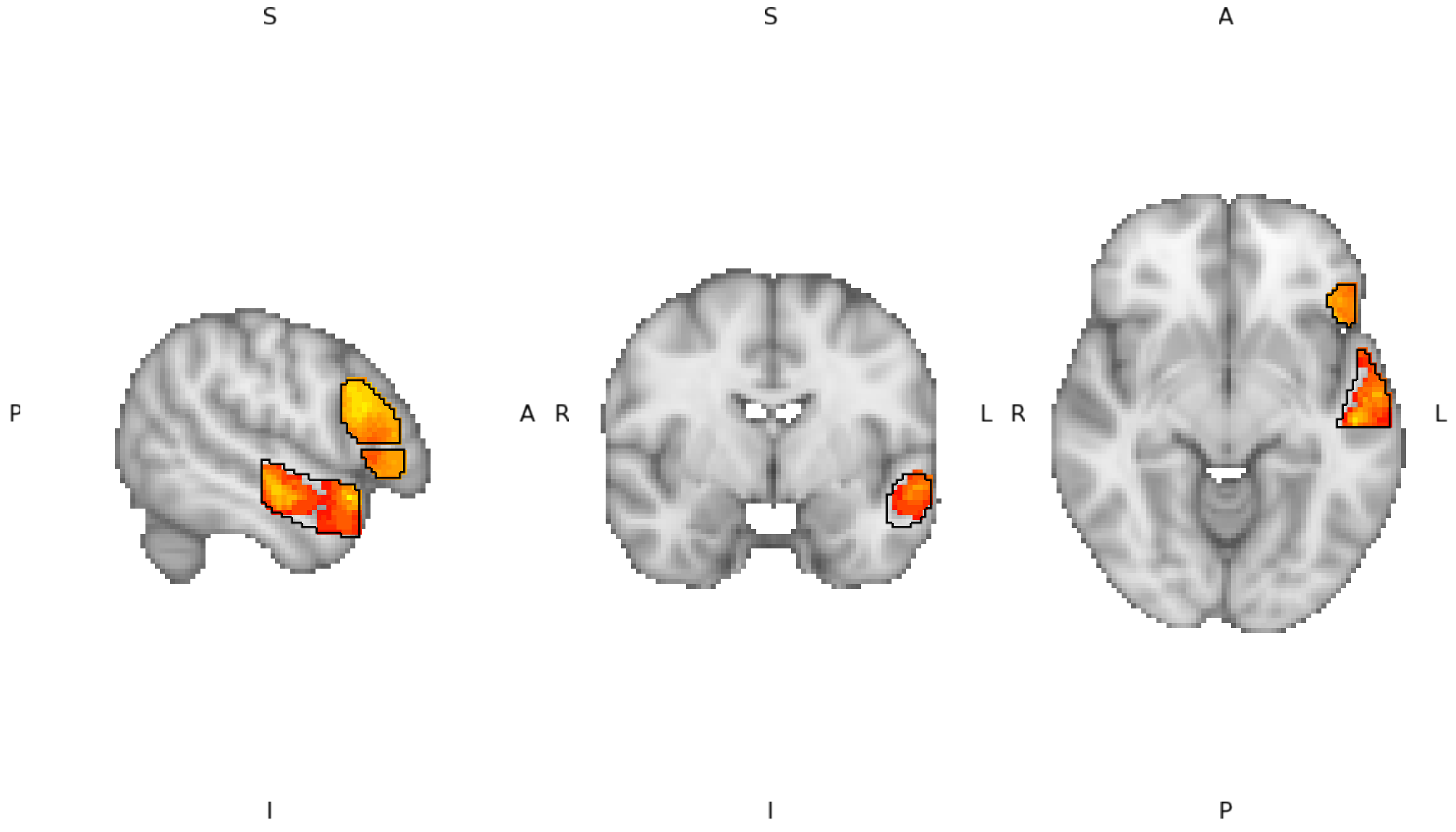 | 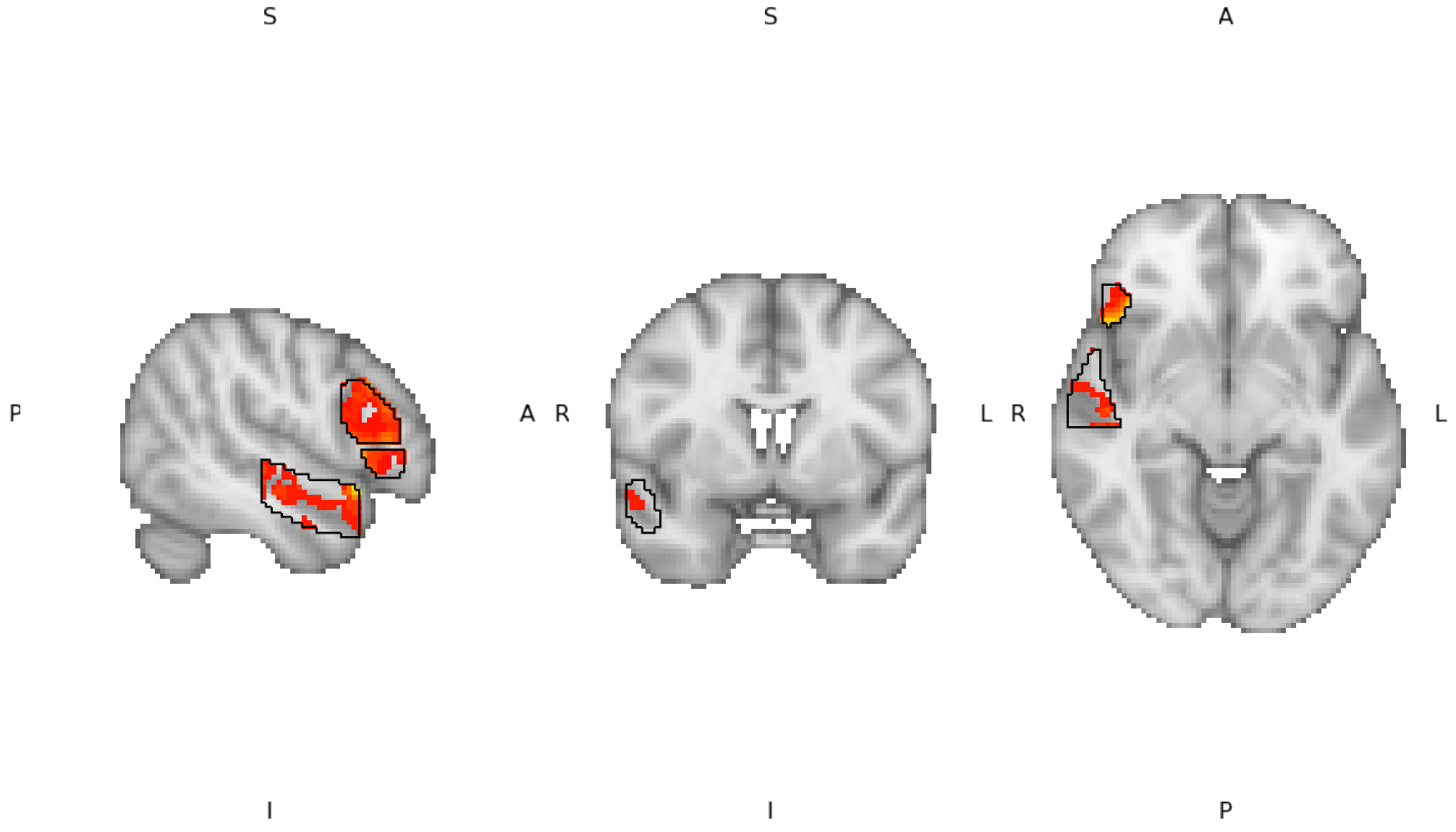 |
| MdLF | STG**  AG** | TP** | 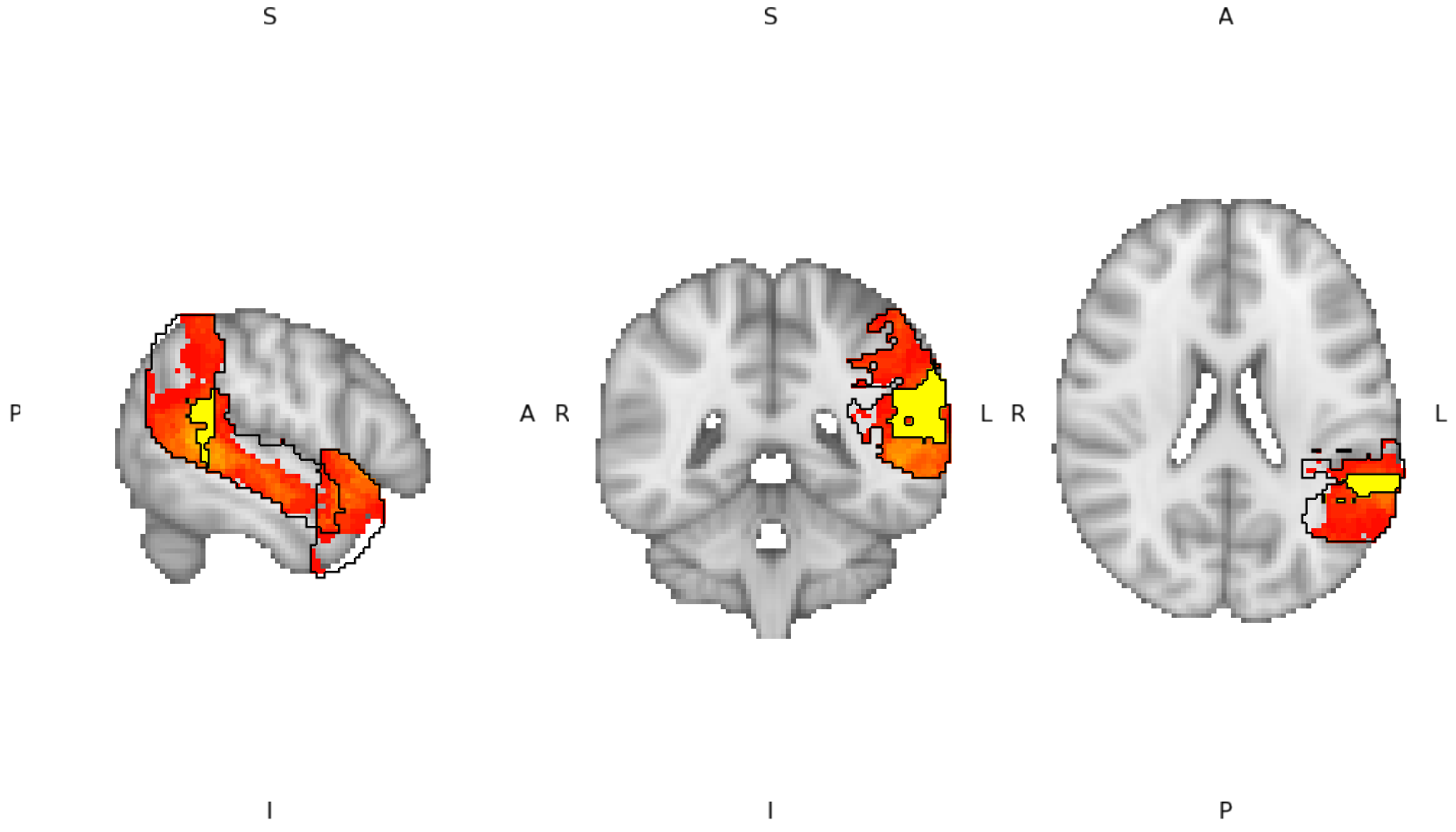 | 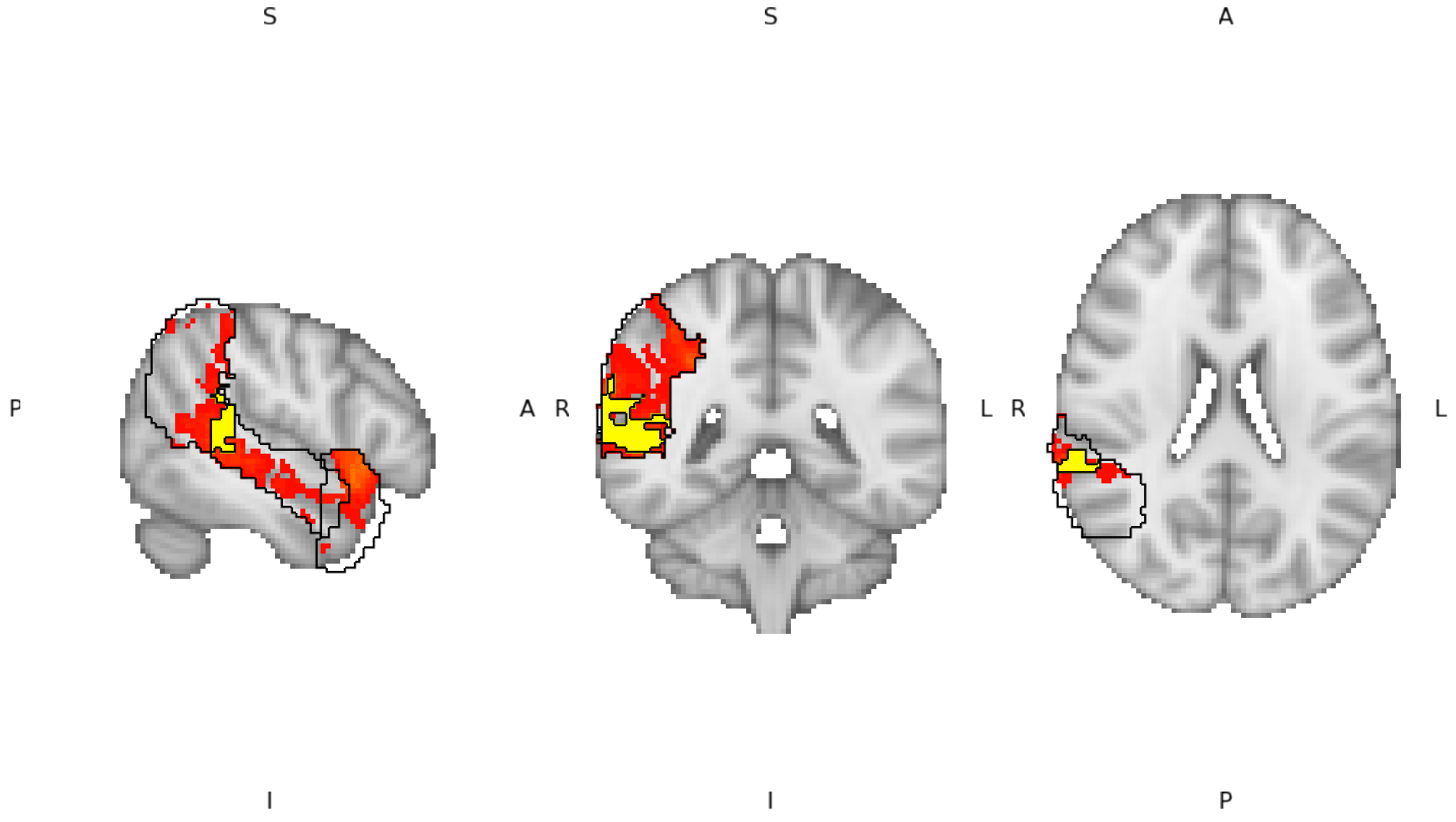 |
| * fMRI-guided partitioning of the cortex [1,2]  ** Harvard-Oxford Cortical Structural Atlas as implemented in FSL | | | | |

| **Hemisphere** | **Tract** | **Algorithm** | **Subject** | **Seed** | **Target** |
| --- | --- | --- | --- | --- | --- |
| Left | SLF3 | Ftract | 12 | - | - |
|  | UF | Ftract | 09  12 | +  + | +  + |
|  | MdLF | Ftract | 12 | + | - |
| Right | FAT | Ctract | 07 | + | + |
|  | FAT | Ftract | 01  07  20 | +  +  + | +  -  - |
|  | AF | Ftract | 07  12  17  20 | -  +  +  - | +  -  +  + |
|  | SLF3 | Ftract | 12  20 | -  - | +  + |
|  | ILF | Ftract | 12  15 | -  + | +  - |
|  | UF | Ftract | 04  15 | +  - | +  + |

**Supplementary Table 8** Failed tract reconstructions

**Supplementary Table 9 Cases with perfect overlay between Xtract and Ctract** (fslcc *r* = 1 Fisher *Z* = Inf.)

| **Hemisphere** | **Tract** | **Correlation** | **Subject** |
| --- | --- | --- | --- |
| Left | UF | Xtract-Ctract | 12 |
| Right | AF | Xtract-Ctract | 07  17 |
|  | ILF | Xtract-Ctract | 01 |
|  | UF | Xtract-Ctract | 04 |

**References**

[1] Mahowald K, Fedorenko E. Reliable individual-level neural markers of high-level language processing: A necessary precursor for relating neural variability to behavioral and genetic variability. Neuroimage 2016;139:74–93. <https://doi.org/10.1016/j.neuroimage.2016.05.073>.

[2] Fedorenko E, Hsieh P-J, Nieto-Castañón A, Whitfield-Gabrieli S, Kanwisher N. New Method for fMRI Investigations of Language: Defining ROIs Functionally in Individual Subjects. J Neurophysiol 2010;104:1177–94. <https://doi.org/10.1152/jn.00032.2010>.
